# Mixtures of l-Amino Acids as Reaction Medium for Formation of Iron Nanoparticles: The Order of Addition into a Ferrous Salt Solution Matters

**DOI:** 10.3390/ijms141019452

**Published:** 2013-09-25

**Authors:** Karolína M. Šišková, Libor Machala, Jiři Tuček, Josef Kašlík, Peter Mojzeš, Radek Zbořil

**Affiliations:** 1 Department of Physical Chemistry, Regional Centre of Advanced Technologies and Materials, Palacký University in Olomouc, 783 71 Olomouc, Czech Republic; E-Mail: radek.zboril@upol.cz; 2 Department of Experimental Physics, Regional Centre of Advanced Technologies and Materials, Palacký University in Olomouc, 783 71 Olomouc, Czech Republic; E-Mails: libor.machala@upol.cz (L.M.); jiri.tucek@upol.cz (J.T.); josef.kaslik@upol.cz (J.K.); 3 Institute of Physics, Faculty of Mathematics and Physics, Charles University in Prague, Prague, Ke Karlovu 5, 121 16 Prague 2, Czech Republic; E-Mail: mojzes@karlov.mff.cuni.cz

**Keywords:** γ-Fe_2_O_3_, FeOOH, zero-valent iron, NZVI, Mössbauer spectroscopy, SERS, arginine, arginate, glutamic acid, glutamate, nanomagnetism

## Abstract

Owing to Mössbauer spectroscopy, an advanced characterization technique for iron-containing materials, the present study reveals previously unknown possibilities using l-amino acids for the generation of magnetic particles. Based on our results, a simple choice of the order of l-amino acids addition into a reaction mixture containing ferrous ions leads to either superparamagnetic ferric oxide/oxyhydroxide particles, or magnetically strong Fe^0^-Fe_2_O_3_/FeOOH core-shell particles after chemical reduction. Conversely, when ferric salts are employed with the addition of selected l-amino acids, only Fe^0^-Fe_2_O_3_/FeOOH core-shell particles are observed, regardless of the addition order. We explain this phenomenon by a specific transient/intermediate complex formation between Fe^2+^ and l-glutamic acid. This type of complexation prevents ferrous ions from spontaneous oxidation in solutions with full air access. Moreover, due to surface-enhanced Raman scattering spectroscopy we show that the functional groups of l-amino acids are not destroyed during the borohydride-induced reduction. These functionalities can be further exploited for (i) attachment of l-amino acids to the as-prepared magnetic particles, and (ii) for targeted bio- and/or environmental applications where the surface chemistry needs to be tailored and directed toward biocompatible species.

## 1. Introduction

Iron nanoparticles belong to the group of magnetic particles which, owing to the presence of magnetic spin states, provide them with magnetic behavior which has been known for centuries [1]. Among iron-containing particles, superparamagnetic ferric oxides (mostly in the form of nanoparticulate maghemite, γ-Fe_2_O_3_) and zero-valent iron nanoparticles (generally labeled as NZVI and/or nZVI) are the most extensively studied because of their applications in magnetic resonance imaging [2] and environmental clean-up applications [3,4], respectively. The global magnetic properties of iron nanoparticles are strongly dependent on their size, composition (in terms of mutual ratios of iron oxidation and spin states), and structural arrangement of iron atoms within the nanoparticles of a particular sample [5–8]. Therefore, it is obvious that iron nanoparticles have to be thoroughly characterized, *i.e.*, their size, exact composition, and structural arrangement of iron atoms within the sample determined. A unique excellent technique to provide those characteristics for iron-containing materials simultaneously is Mössbauer spectroscopy [5–12]. Unfortunately, this technique is not widespread or well-known for characterizing iron nanoparticles (e.g., [13–16]), although it is the most suitable and adequate one. With the present work, we would like to point out and demonstrate the potential of Mössbauer spectroscopy in iron nanoparticles investigation.

On studying the literature, the combination of iron nanoparticles with l-amino acids, poly(amino acids), oligopeptides and/or proteins is extensive [8,9,16–27], however reasons for investigation vary significantly. In some cases, the mode of, strength of, and conditions for the adsorption of proteins [24,25], oligopeptides [21,22] or different single amino acids [19,26] on iron oxide nanoparticle surfaces are of major interest. Conversely, the Jong-Duk Kim group [20] developed a method to transfer hydrophobic magnetite nanoparticles from organic solvents into water using a poly(amino acid) derivative with a hydrophilic backbone and side chains. Furthermore, iron oxide mineralization in the presence of different single l-amino acids of various concentrations was investigated by Mantion and co-workers [18]. Additional research groups investigated the precipitation of iron oxides particles either in the presence [16,17], or directly induced by single l-amino acids [27].

From previous literature, it is thus obvious that mainly the surfaces of iron oxide nanoparticles have been modified by single l-amino acids (bio-compatible forms of amino acids) and/or their natural (oligopeptides, proteins) or artificial polymeric variants either directly during nanoparticle syntheses, or subsequently (post-modification). It should be noted that very little (except for two papers published by us very recently [8,9]) is known about the influence of single l-amino acids present in the course of nanoscale zero-valent iron (NZVI) formation. However, in our previous reports about NZVI formation in the presence of single l-amino acids [8,9], we did not investigate and explain the reason why l-amino acids behave differently in ferrous and ferric ions solutions. Furthermore, we gave no evidence in refs. [8,9] about the distinct differences in the final iron particles composition when mixtures of l-amino acids were used.

Indeed, in ref. [8], we explored the ability of l-glutamic acid (Glu) to act as an efficient mediator of NZVI formation. For the choice of Glu we were inspired by other authors [16–19,27] who had demonstrated a direct chemical bonding (through carboxylic group/s) of carboxylic acids to the surface of iron oxide nanoparticles. Since iron oxide is always present around a zero-valent iron core (when working under ambient conditions), we hypothesized [8] that a secondary organic shell could be created by amino acids which would improve the interaction with halogeno-organic pollutants. This hypothesis was evidenced and further demonstrated on trichloroethane removal [8]. According to our results [8], the atomic percentage of zero-valent iron generated in the presence of Glu was about 10% higher than in the case without Glu (and without any other l-amino acid) being present, in the course of ferrous sulphate reduction by sodium borohydride.

Conversely, in ref. [9], we compared the influence of four selected l-amino acids and their pH-dependence on NZVI formation. As derived from the results in ref. [9], the pH value of the reaction mixture before borohydride addition has a strong effect on the final Fe^0^:Fe^3+^ ratio when acidic l-amino acids are used. On the contrary, if basic l-amino acids are employed as reaction mediators, there is no distinct dependence of the final Fe^0^:Fe^3+^ ratio on the pH value of the reaction mixture [9].

Therefore, based on our previous results, we have investigated in the present work the influence of l-arginine (Arg) as a mediator of NZVI formation, as well as, the effect of mixtures of Glu and Arg on NZVI generation from ferrous and/or ferric salts. Arg was intentionally chosen for the reason that it possesses a guanidine function, having a very strong basic character. Moreover, it can react with ferrous chloride, thus resulting in maghemite and/or magnetite as described in the literature [27]. Using several different characterization techniques such as Mössbauer spectroscopy, X-ray powder diffraction (XRD), transmission electron microscopy (TEM), we therefore looked into possible iron particles formation when the above mentioned l-amino acids were subsequently introduced. To the best of our knowledge, nobody has yet investigated the effect of mixtures of l-amino acids on NZVI formation from ferrous and/or ferric salts. Moreover, employing SQUID (superconducting quantum interference device) magnetometry, we also measured the global magnetic behavior of the as-prepared iron nanoparticles generated in the presence of the Glu and Arg mixtures. We discussed the global magnetic properties in conjunction with the interpretation of Mössbauer spectra. Furthermore, the stability of iron nanoparticles was checked after two years of storage. Finally, using surface-enhanced Raman spectroscopy (SERS, exploiting pre-prepared silver nanoparticles), we also observed no changes in Arg and Glu when these l-amino acids were allowed to react with sodium borohydride at the particular concentration used throughout our syntheses. This important point has never been addressed in the currently available literature.

## 2. Results and Discussion

Prior to the investigation using l-amino acid mixtures on NZVI formation, it was necessary to investigate the effect of each l-amino acid when applied separately to ferrous and/or ferric salt and to evaluate changes in reactant structures in the concentrations simulating the reaction medium. Therefore, the results of our experiments are discussed in the following text divided into four sections dealing with (i) Arg *vs.* Glu mediating NZVI formation from ferrous and/or ferric salts (the samples named Fe2ArgBH, Fe2GluBH, Fe3ArgBH and Fe3GluBH, respectively); (ii) interaction between Arg and/or Glu with ferrous sulphate dissolved in solution (the samples labeled as Fe2Arg and/or Fe2Glu, respectively); (iii) influence of sodium borohydride (a strong reducing agent) on Arg and/or Glu investigated by SERS (the samples named ArgBH and/or GluBH, respectively); (iv) impact of l-amino acids mixtures on NZVI generation from ferrous and/or ferric salts (the samples labeled as Fe2ArgGluBH, Fe2GluArgBH, Fe3ArgGluBH and/or Fe3GluArgBH). Finally, the stability of iron particles after two years of storage is verified by means of Mössbauer spectroscopy and their nanoparticulate character is also investigated by TEM.

### 2.1. Single l-Amino Acid Mediating NZVI Formation

Based on our recent successful results in NZVI formation mediated by Glu [8], we decided to investigate the effect of another l-amino acid, namely arginine. This basic l-amino acid was intentionally chosen for the fact that it (a) bears not only an amino moiety, but also a guanidyl moiety in its structure; and (b) can react with ferrous salts resulting in magnetic iron oxide nanoparticles as claimed in the literature [27]. However, in the literature mostly XRD is used for nanoparticles characterization and determination of phases included in a sample. The usage of XRD is misleading in many cases (e.g., [13–16]). In fact, the results obtained by diffraction techniques are related to crystalline parts of the sample only and cannot detect any amorphous material [5] or very thin layers on the surface of nanoparticles [8]. There is, however, an excellent tool for the investigation of iron-containing samples, *i.e.*, Mössbauer spectroscopy. Since we worked exclusively with iron nanoparticles in our study, we fully exploited the potential of this iron-sensitive technique in order to characterize our samples and correctly evaluate the influence of each l-amino acid added into the reaction mixture.

**Figure 1:**
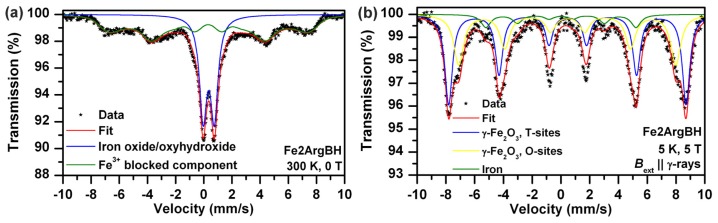

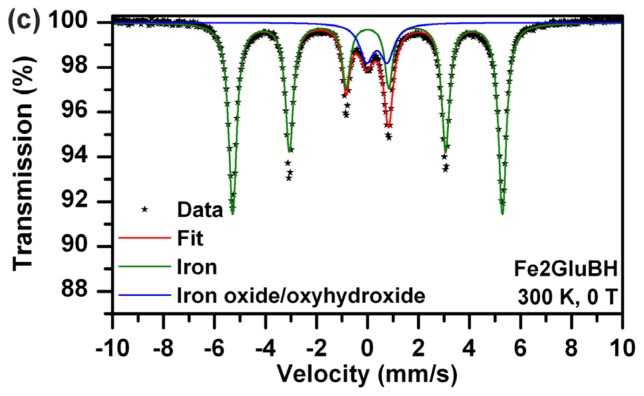
(**a**) Mössbauer spectrum of Fe2ArgBH recorded at room temperature; (**b**) Mössbauer spectrum of Fe2ArgBH recorded at 5 K; (**c**) Mössbauer spectrum of Fe2GluBH recorded at room temperature.

Direct comparison of Mössbauer spectra of Fe2ArgBH (Figure 1a,b) and Fe2GluBH (Figure 1c) revealed distinct differences in sample composition (Table 1). In the case of Fe2ArgBH two spectra of the same sample are shown in Figure 1; the former is recorded at room temperature (Figure 1a) and the latter at 5 K (Figure 1b). The Mössbauer spectrum of Fe2ArgBH measured at room temperature (Figure 1a) is fitted with two spectral components, one doublet and one sextet. However, the sextet has a very broad line width which points to a distribution of hyperfine magnetic fields. In order to distinguish the exact composition of Fe2ArgBH, a Mössbauer spectrum at 5 K had to be measured (Figure 1b). The experimental data points in Figure 1b could be fitted with three components: three sextets with different isomer shifts (IS), quadrupole splitting (QS), and hyperfine magnetic field values. Two of these sextets (IS of 0.48 and 0.39 mm/s, QS of −0.02 and −0.03 mm/s, hyperfine magnetic field of 46.9 and 51.3 T) were attributed to ferric ions in octahedral and tetrahedral positions of γ-Fe_2_O_3_, respectively (Table 1) [7]. The third sextet (IS of 0.10 mm/s, QS of 0.00 mm/s, hyperfine magnetic field of 32.4 T) was assigned to α-Fe [28]. The areas below each sub-spectrum revealed that 90% of iron atoms in Fe2ArgBH correspond to gamma ferric oxide, while only 10% of iron atoms are found in the zero oxidation state. This is a completely reversed percentual composition from that obtained for Fe2GluBH (Figure 1c), where ~90% of iron atoms are found in the zero valence state and ~10% of iron atoms in gamma ferric oxide (Table 1).

**Figure 2:**
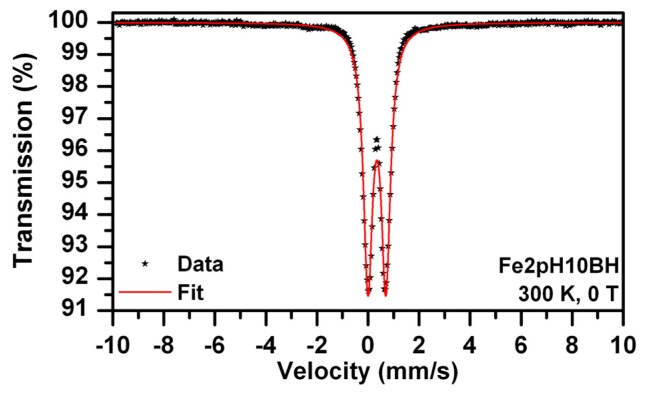
Mössbauer spectrum of Fe2pH10BH recorded at room temperature.

It should be noted that the preparation of both systems was performed in exactly the same way and under the same experimental conditions. Therefore, the only difference lies in the presence of either Arg, or Glu. Arg is known to induce alkaline pH values of the solutions [27]. Indeed, the pH value of 10 mM Arg solution was measured by us and recorded at 10.02. We consequently prepared a solution of the same pH value employing sodium hydroxide and used it for the dissolution of ferrous sulphate followed by reduction induced by sodium borohydride (sample named Fe2pH10BH). The concentrations of FeSO_4_ and NaBH_4_ were adjusted to the same as in the case of Fe2ArgBH. The Mössbauer spectrum of the resulting system is shown in Figure 2 and the parameters of the fit listed in Table 1. Only a doublet (IS of 0.35 mm/s, QS of 0.70 mm/s) can best fit the experimental data points (Figure 2). This doublet can be attributed to gamma ferric oxide/oxyhydroxide [7].

**Scheme 1:**
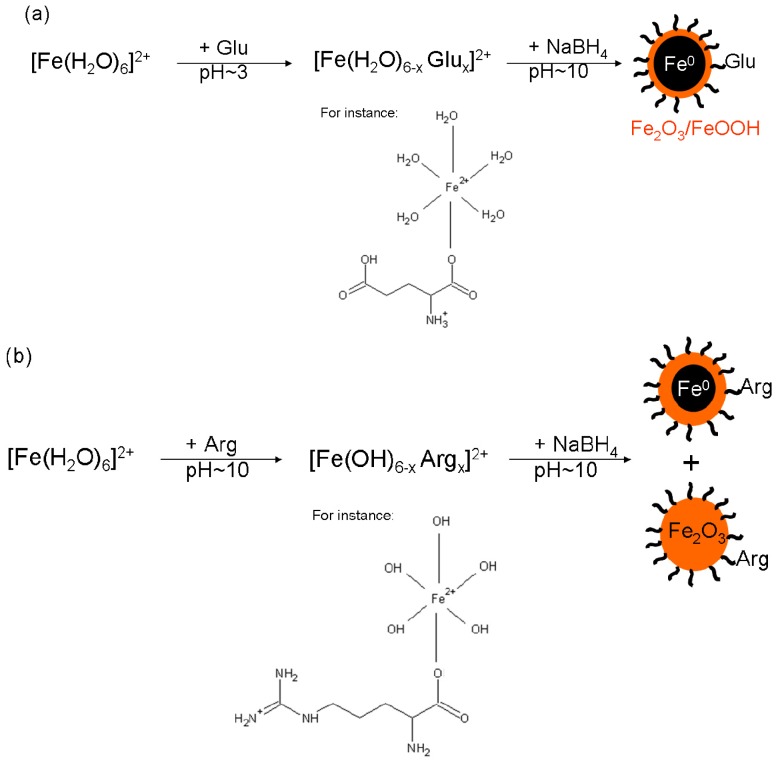
Depiction of intermediate complexes formed by Glu (**a**) and/or Arg (**b**) with ferrous salt dissolved in solution and reduced by NaBH_4_ in the next step. Hypothetical structures of intermediate complexes (at pH 3 for Glu-FeSO_4_ and at pH 10 for Arg-FeSO_4_) are suggested. Resulting types of iron nanoparticles are also schematically depicted.

Thus it can be concluded that alkaline pH values around 10 of the mixture of Arg and FeSO_4_ provide good conditions for the formation of ferric oxide/oxyhydroxides. Subsequent addition of NaBH_4_ (the final concentration of 0.12 M) induced an incomplete reduction of ferric oxide/oxyhydroxides to Fe^0^. Although sodium borohydride itself generated pH~10 in each reaction mixture, the dissolution of ferrous sulphate in an alkaline solution (without reduction ability) led to the formation of ferrous hydroxide in the very first step, which was then oxidized to ferric hydroxide within five minutes due to the access of ambient air. This oxidation induced by air access was further supported by a direct visible observation of solution color changes: the addition of FeSO_4_ into Arg solution resulted in a green precipitate formation which turned brownish orange within five minutes. Therefore, the formation of Fe^0^ was hindered in the case of Fe2ArgBH in comparison to Fe2GluBH due to the generation of ferric oxides/oxyhydroxides which were hardly reduced to Fe^0^ when 0.12 M final NaBH_4_ concentration used. However, taking into account the resulting compositions of Fe2ArgBH (~90% of ferric oxides and ~10% of Fe^0^) and that of the sample presented in Figure 2 (100% of ferric oxide/oxyhydroxides), there is not only the pure effect of alkaline pH value caused by Arg in Fe2ArgBH. We thus hypothesized that an intermediate complex between a particular l-amino acid (partially deprotonated at a given pH value) and ferrous sulphate is formed in the solution. The complex can be reduced by sodium borohydride in the next step of the NZVI formation procedure as is schematically expressed in Scheme 1.

**Figure 3:**
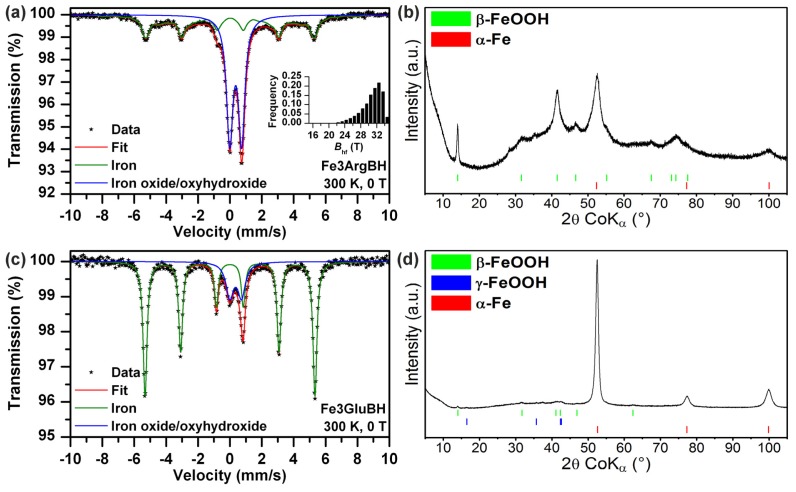
(**a**) Mössbauer spectrum of Fe3ArgBH recorded at room temperature; (**b**) X-ray powder diffraction (XRD) patterns of Fe3ArgBH; (**c**) Mössbauer spectrum of Fe3GluBH recorded at room temperature; (**d**) XRD patterns of Fe3GluBH.

In order to avoid the spontaneous oxidation of iron salt in amino acid solutions with air access, we performed the same type of experiments with the only exception: ferric (instead of ferrous) salt being used as the iron source. The as-prepared Fe3ArgBH and Fe3GluBH were characterized by Mössbauer spectroscopy and for a direct comparison by XRD (Figure 3). According to Mössbauer spectra evaluation, there are two phases: Fe^0^ and Fe^3+^. The quantification of each iron phase based on Mössbauer spectra, revealed that the content of Fe^0^ is much higher in the case of Fe3GluBH than Fe3ArgBH (Table 1). This supports the idea of a specific intermediate complex formation between a particular l-amino acid and iron-containing salt, as well as, the impact of l-amino acid induced pH value changes.

Similar to Mössbauer spectra, XRD data confirmed the presence of Fe^3+^ and Fe^0^ phases as shown in Figure 3. However, the quantification of all phases detectable by XRD revealed 52% of Fe^0^ in Fe3ArgBH (in comparison to 43% based on Mössbauer data–Table 1), with 96% of Fe^0^ in Fe3GluBH (80% according to Mössbauer data–Table 1). It is obvious that there are huge discrepancies between XRD-based (Figure 3) and Mössbauer-based (Table 1) quantifications for the same sample. It stems from the fact that any amorphous phase and/or thin layer of ferric oxide/oxyhydroxides around the nanoparticle Fe^0^ core are practically invisible to XRD (they contribute to the “XRD”-amorphous background); while detected by Mössbauer spectroscopy (as it is very sensitive in distinguishing iron-containing phases when iron nuclei experience different hyperfine interactions due to different local surroundings). In this regard, Mössbauer spectroscopy is much more accurate in solely characterizing iron-containing particles.

### 2.2. Influence of Arg and/or Glu on Ferrous Ions in Solution

**Figure 4:**
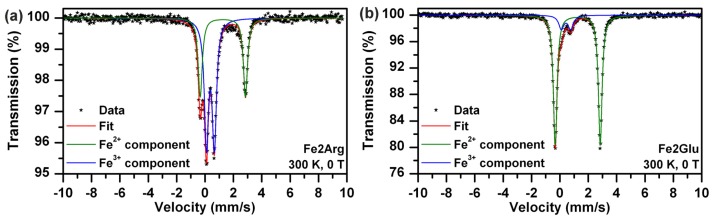
(**a**) Mössbauer spectrum of Fe2Arg recorded at room temperature; (**b**) Mössbauer spectrum of Fe2Glu recorded at room temperature.

In order to demonstrate the effect of Arg and/or Glu acting as mediators of NZVI formation and to evaluate the extent of ferrous to ferric ions oxidation when a particular l-amino acid is present in the solution with full air access enabled, we prepared the samples Fe2Arg and Fe2Glu. Their Mössbauer spectra are compared in Figure 4.

Performing experimental data fits and considering the above mentioned parameters, there were ~62% of ferric and ~38% of ferrous atoms in Fe2Arg (Figure 4a, Table 1); while ~87% of ferrous and only ~13% of ferric ions were detected in Fe2Glu (Figure 4b, Table 1). These results corroborated the effects and consequences of pH increase (in the case of Fe2Arg) and air access (in both cases) on ferrous ions in a solution of l-amino acids.

### 2.3. Effect of Sodium Borohydride on Arg and/or Glu–Verification Based on SERS Measurements

**Figure 5:**
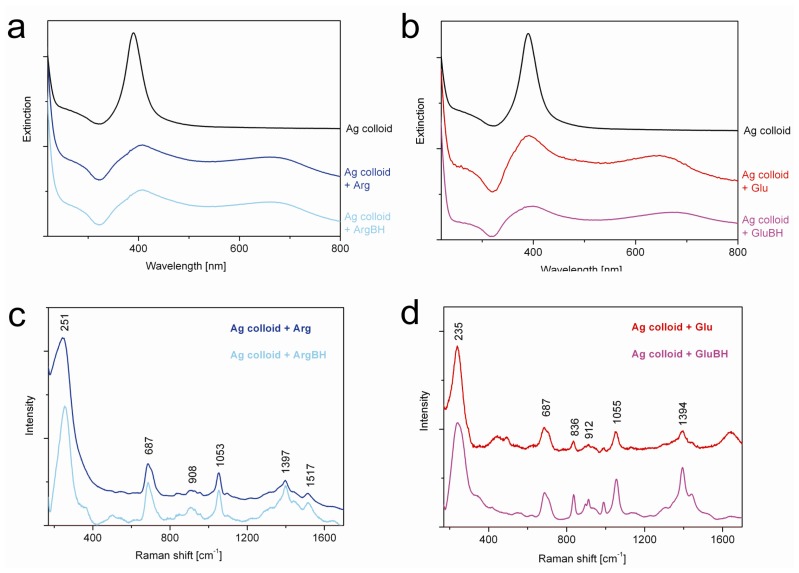
(**a**) Surface plasmon extinction (SPE) spectra of Ag colloid without and with Arg and/or ArgBH; (**b**) SPE spectra of Ag colloid without and with Glu and/or GluBH; (**c**) SERS spectra of Ag colloid with Arg and/or ArgBH; (**d**) SERS spectra of Ag colloid with Glu and/or GluBH.

Taking into account the fact that a strong reducing agent was employed during our syntheses of iron nanoparticles, a question concerning a potential destruction of the structure of the l-amino acids arose. Namely, it is crucial to avoid and/or at least to assess a possible reduction (driven by sodium borohydride) of carboxylic groups in l-amino acids (amino groups are presumed not to be any further reduced). The carboxylic group is well-known for its ability to bind to an iron oxide nanoparticle surface [16–19,27] and/or core-shell Fe^0^-Fe_2_O_3_/FeOOH nanoparticles [8]. Therefore, we decided to check whether the carboxylic functional groups of Arg and/or Glu were preserved when they were allowed to react with sodium borohydride. The samples called ArgBH and GluBH were prepared, respectively. Since the concentration of a particular l-amino acid was low in comparison to that of borate (which arises from sodium borohydride), regular methods of vibrational spectroscopy such as infrared absorption and/or Raman scattering could not be used (borate signal prevailed). Another method, which enables selective and sensitive detection at low concentrations, was chosen, *i.e.*, SERS [29–31]. Due to the possibility of a direct covalent interaction of the amino and/or carboxylic group [32,33] of the l-amino acids with Ag nanoparticle surfaces, it was presumed that the enhancement of the Raman signal would be much stronger for l-amino acids than for borates interacting through electrostatic bonds with Ag nanoparticle surfaces. Similarly as in the case of our recent study [34], we thus exploited SERS in conjunction with surface plasmon extinction (SPE) spectroscopy for monitoring changes in l-amino acids used in this study. Figure 5 shows the resulting SPE and SERS spectra.

At first, SPE spectra (Figure 5a,b) were measured in order to determine the most suitable wavelength of a continuous laser for the excitation of Ag nanoparticles interacting with l-amino acids and/or ArgBH/GluBH. Usually surface plasmon of non-interacting silver nanoparticles in aqueous solution appears at ~400 nm [29,30]. However, this characteristic feature of Ag nanoparticles is red-shifted when mutual interactions and consequent aggregation of Ag nanoparticles come into play. In our cases (Figure 5a,b), the second maximum in the SPE spectra arose at ~660 nm. This can be related to Ag nanoparticles aggregation induced by Arg and/or ArgBH in Figure 5a, whereas by Glu and/or GluBH in Figure 5b. The closest excitation wavelength available for us was 633 nm which was thus employed and the resulting SERS spectra recorded (Figure 5c,d).

For the sake of a direct comparison, the SERS spectra were normalized with respect to the most intensive peak and off set in Figure 5c,d. It is obvious that positions and relative intensities of all peaks within each SERS spectrum of Arg and/or ArgBH interacting with silver nanoparticles (Figure 5c) are virtually the same. This gives evidence that all important functionalities of Arg were preserved and no changes caused by sodium borohydride (employed in huge abundance: 60 NaBH_4_*vs.* 1 amino acid in the sample named as ArgBH and/or GluBH). Based on the SERS spectra presented in Figure 5d, exactly the same statement can be adopted for Glu and GluBH. Now recall that only the Raman signal of Arg and/or Glu molecules close to the silver nanoparticle surface can be enhanced. In other words, NaBH_4_ did not reduce the carboxylic groups of the particular l-amino acids in our systems.

Moreover, SERS measurements revealed the availability of amino and carboxyl groups in ArgBH and GluBH, respectively, for the interaction with the silver nanoparticle surface. Namely, the most intensive peak positioned at ~250 cm^−1^ observed in Figure 5c corresponds most probably to Ag-N interaction [32]. Conversely, the most intensive peak in Figure 5d, positioned at ~235 cm^−1^, can be assigned to Ag-OOC bond [33]. This means, in turn, that both functional groups being present in ArgBH and GluBH can be exploited for the interaction either with iron nanoparticle surfaces (in the present work), or with any other type of suitable molecule and/or nanoparticle from solution. This is particularly important for further surface modifications of iron nanoparticles, decorated by l-amino acids on their surfaces, with the aim to tailor them for a specific application.

### 2.4. Impact of l-Amino Acids Mixtures on NZVI Generation

**Figure 6:**
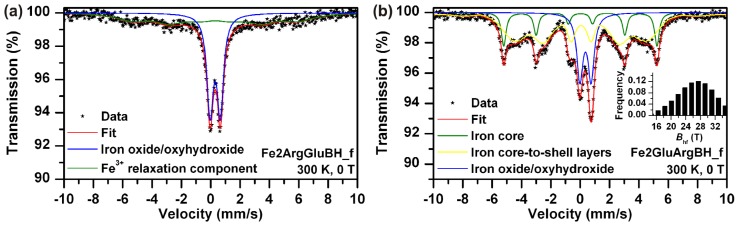

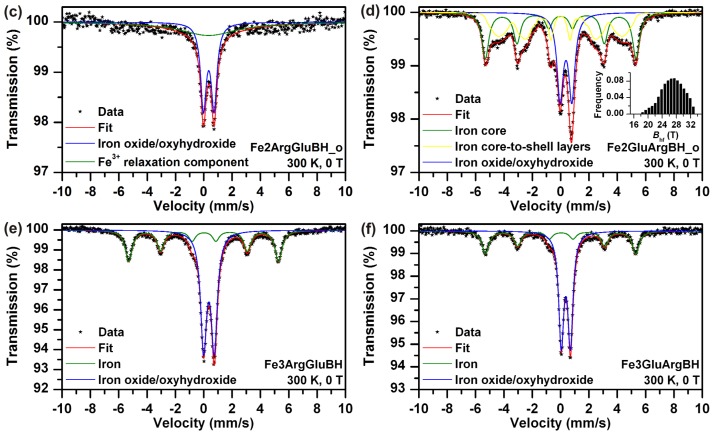
Mössbauer spectra of (**a**) Fe2ArgGluBH freshly prepared, recorded at room temperature; (**b**) Fe2GluArgBH freshly prepared, recorded at room temperature; (**c**) Fe2ArgGluBH two-years aged, recorded at room temperature; (**d**) Fe2GluArgBH two-years aged, recorded at room temperature; (**e**) Fe3ArgGluBH, recorded at room temperature; (**f**) Fe3GluArgBH, recorded at room temperature.

**Figure 7:**
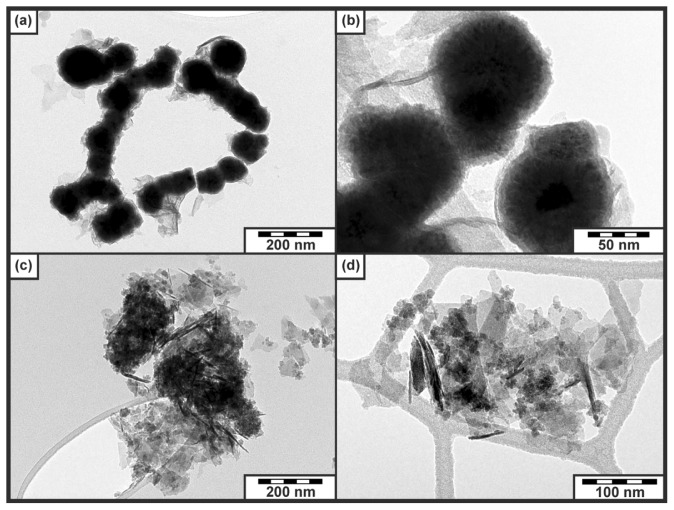
TEM images of (**a**,**b**) Fe2GluArgBH and (**c**,**d**) Fe2ArgGluBH.

**Figure 8:**
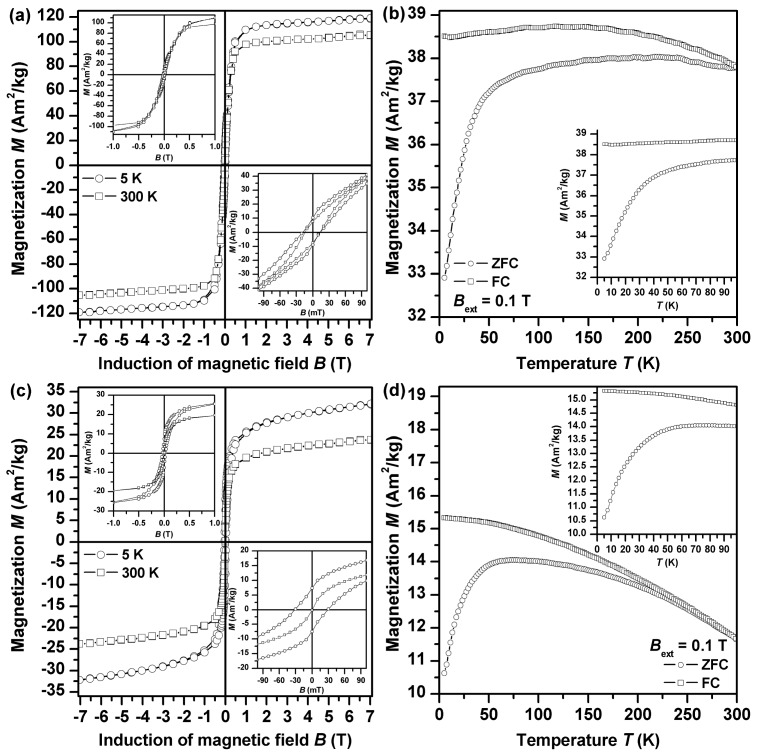
(**a**) 5 K and room-temperature hysteresis loops of Fe2GluArgBH; (**b**) ZFC (zero-field-cooled) and FC (field-cooled) magnetization curves of Fe2GluArgBH; (**c**) 5 K and room-temperature hysteresis loops of Fe2ArgGluBH; (**d**) ZFC and FC magnetization curves of Fe2ArgGluBH.

Since the effect of each l-amino acid used in this study had already been discussed and evaluated, the impact of addition order was also investigated. Basically two different approaches could be applied: (a) dissolution of ferrous sulphate in Arg solution and subsequent addition of Glu or (b) dissolution of ferrous sulphate in Glu solution and subsequent addition of Arg. We performed both, followed by immediate reduction (within five minutes after mixture preparation), driven by sodium borohydride (the 0.12 M final concentration). The resulting systems were characterized by Mössbauer spectroscopy, TEM imaging, and SQUID magnetometry. Mössbauer spectra are shown in Figure 6, TEM images in Figure 7, and SQUID measurements data in Figure 8.

While Fe2ArgGluBH (Figure 6a) consists of superparamagnetic and magnetically ordered Fe_2_O_3_/FeOOH particles, thus only high spin ferric phase (Table 1); Fe2GluArgBH (Figure 6b) contains mostly Fe^0^ (79 at.% from which 63 at.% corresponded to rather small particles) and minor Fe_2_O_3_ phase (21 at.%). Therefore, it can be concluded that the choice of the first l-amino acid interacting with ferrous ions in solution critically influences the resulting phase composition of the whole sample. In other words, it is the determining step of the final iron oxidation state: Glu promotes Fe^0^ formation, whereas Arg leads toward superparamagnetic Fe_2_O_3_/FeOOH particles when subsequent chemical reduction is performed. Furthermore, an intermediate complex between Glu and Fe^II^ forms (Scheme 1a) and survives, to some extent, even under alkalization of the system by the dissolution of Arg.

Importantly, both samples (Fe2ArgGluBH and Fe2GluArgBH) manifested themselves by a bimodal distribution of particle sizes as derived from Mössbauer data (Figure 6a,b) and confirmed by TEM imaging (Figure 7): (i) big particles of Fe_2_O_3_/FeOOH being magnetically ordered (Figure 6a) and/or big particles of Fe^0^ providing a well-defined sextet with narrow lines (Figure 6b); (ii) small particles of Fe_2_O_3_/FeOOH revealing superparamagnetic behavior (Figure 6a) and/or distribution of small particles of Fe^0^ (Figure 6b). The bimodal distribution of particle sizes can be related to the simultaneous effect of both l-amino acids present in reaction mixtures because such a phenomenon (bimodal distribution) was not observed in the cases when each l-amino acid was applied separately.

Furthermore, TEM images clearly visualized the core-shell structure of nanoparticles in the case of Fe2GluArgBH (Figure 7a,b). Conversely, very small nanoparticles (with no core-shell structure) together with needle-like structures and/or plates were observed in the case of Fe2ArgGluBH (Figure 7c,d). While the former is very similar to the characteristic TEM images of Fe2GluBH published in ref. [8]; the latter resembles TEM features of FeOOH and/or amorphous Fe_2_O_3_[5,27]. There is thus a distinct difference in the order of l-amino acids addition to ferrous salt on the resulting iron-containing nanoparticulate structures. This conclusion can be made not only by considering TEM images, but also taking into account Mössbauer and SQUID data.

The global magnetic properties of both samples were monitored by measurement of their field- and temperature-dependent magnetization (Figure 8). In all measuring regimes performed, magnetization values are much higher for Fe2GluArgBH (Figure 8a,b) than those observed for Fe2ArgGluBH (Figure 8c,d). This is to be expected as Fe^0^ gives a much stronger magnetic response than γ-Fe_2_O_3_/FeOOH. At 5 K, the isothermal magnetization curve of both Fe2GluArgBH and Fe2ArgGluBH samples shows a hysteresis character (Figure 8a,c), indicating a magnetically ordered state to which nanoparticles entered on cooling. In the case of Fe^0^ nanoparticles, the magnetically ordered state is of a ferromagnetic character whereas for γ-Fe_2_O_3_ nanoparticles, the blocked state of their particle magnetic moments is supposed to evolve at low temperature. For the Fe2GluArgBH sample, a steep change in magnetization values is observed around the origin of the hysteresis loop (inset in Figure 8a), implying a presence of some impurity with magnetic response distinct from Fe^0^. On the other hand, no such behavior of the hysteresis loop trend is detected for Fe2ArgGluBH (inset in Figure 8c), confirming, within the experimental error of the SQUID technique, its single-phased nature. This is in accordance with the phase composition of studied samples derived from the analysis of the Mössbauer spectra. In addition, the 5 K hysteresis loop of Fe2GluArgBH (inset in Figure 8a) shows asymmetry (*i.e.*, difference in the values of the positive, *B*_C+_, and negative, *B*_C−_, coercivity–*B*_C+_ = 13.6 mT and *B*_C−_ = 18.2 mT), a typical feature by which an exchange bias phenomenon is manifested [35]. Exchange bias phenomenon frequently develops when an interface between two phases with magnetic ordering distinct from each other is formed. Beside other systems, this is commonly observed for nanoparticles having core-shell architecture [36]. In our case, the particle core is made up of Fe^0^ with ferromagnetic ordering whereas the particle shell is of γ-Fe_2_O_3_/FeOOH nature showing a ferrimagnetic alignment partly distorted by surface effects. The presence of a γ-Fe_2_O_3_/FeOOH shell is demonstrated by enhancement in coercivity values (for bulk and pure iron, the coercivity values are frequently smaller than 0.1 mT [37]). A core-shell character of nanoparticles in Fe2GluArgBH is also in part supported by a magnetization value under +7 T (~119 Am^2^/kg) [37], which is reduced compared to the saturation magnetization of bulk iron (~220 Am^2^/kg) [37]. Beside this, the lowering of magnetization values is caused by the presence of diamagnetic l-amino acids as magnetization values are recalculated on the total weight of the sample measured. On the other hand, for Fe2ArgGluBH, no asymmetric trend of the 5 K hysteresis loop (inset in Figure 6c) is observed (*B*_C+_ = *B*_C−_ = 30.0 mT), indicating no interface between the magnetically distinct phases. Comparing the coercivity value of γ-Fe_2_O_3_ nanoparticles in the Fe2ArgGluBH sample with that of bulk γ-Fe_2_O_3_ (~25 mT) [37], a slight increase can be attributed to the evolution of finite-size and surface effects modifying (strengthening) the particle magnetic anisotropy [38]. Their presence can also contribute to a reduction of a magnetization value under +7 T when comparing it with the saturation magnetization of bulk Fe_2_O_3_ (~85 Am^2^/kg for γ-Fe_2_O_3_) [37]. Similarly, as for Fe2GluArgBH, diamagnetic l-amino acids play a significant role in lowering the magnetization values.

At 300 K, the profile of the hysteresis loops measured for Fe2GluArgBH and Fe2ArgGluBH markedly differ (Figure 8a,c, respectively). While hysteretic behavior is still observed for Fe2GluArgBH (inset in Figure 8a), Fe2ArgGluBH (inset in Figure 8c) shows no hysteresis. This implies that γ-Fe_2_O_3_/FeOOH nanoparticles irrespective of their size in the assembly have entered into the superparamagnetic state during the time scale of SQUID measurements (~10 s). On the other hand, Fe^0^-Fe_2_O_3_ core-shell nanoparticles are still in a magnetically stable regime with their magnetic moments fixed along the characteristic easy axis of magnetization. Note that no asymmetry in the hysteresis loop of Fe2GluArgBH (inset in Figure 8a) is detected; some of the phases present must have entered into a (super)paramagnetic state on warming.

To get a deeper insight into the magnetic behavior of nanoparticles exhibited in both systems studied, zero-field-cooled (ZFC) and field-cooled (FC) magnetization curves were recorded (Figure 8b,d). It is known that ZFC and FC magnetization curves are very sensitive to magnetic relaxation phenomena such as superparamagnetism. In the case of Fe2GluArgBH (Figure 8b), the slope of the ZFC magnetization curve changes rapidly above ~50 K, implying the onset of a transition of γ-Fe_2_O_3_ phase to superparamagnetic regime (superparamagnetism of Fe^0^ is expected for much smaller nanoparticles, <5 nm). This disconnects the magnetic interaction between the particle core and shell breaking the exchange bias phenomenon (no asymmetry in the hysteresis loop observed, see above). On the other hand, for Fe2ArgGluBH (Figure 8d), two features are identified on the ZFC and FC magnetization curve: (i) a maximum in the ZFC magnetization curve (at ~60 K); and (ii) divergence of the ZFC and FC magnetization curve at ~240 K—both features typical for the transition from the blocked to superparamagnetic regime on warming. While the maximum in the ZFC magnetization curve corresponds to the blocking temperature of nanoparticles with the most probable size in the assembly, the temperature at which ZFC and FC magnetization curves separate (the so-called irreversible temperature) reflects the onset of the blocking mechanism of magnetic moments belonging to the biggest nanoparticles in the system. Hence, the difference in the blocking and irreversible temperature can be regarded as a measure of particle size distribution. This is in accordance with the analysis of the Mössbauer spectra, where the coexistence of sextet and doublet components, indicating a particle size distribution, was observed. At 300 K, ZFC and FC magnetization curves overlap as expected in a superparamagnetic state (no hysteresis in the room-temperature isothermal magnetization curve).

The stability of Fe2ArgGluBH and Fe2GluArgBH stored in closed vials under regular laboratory conditions for two years was investigated. Mössbauer spectra of the samples labeled as Fe2ArgGluBH_o and Fe2GluArgBH_o are presented in Figure 6c,d, respectively, the parameters derived from the spectra are summarized in Table 1. Comparing the spectral features of both samples measured as fresh (Figure 6a,b) and re-measured after two years (Figure 6c,d), they appear similar. However, regarding the parameters of spectral fits (Table 1), the samples slightly changed. Fe2ArgGluBH_o contained a singlet corresponding to a ferric relaxation component together with the doublet of ferric oxide/oxyhydroxide shell. The occurrence of the ferric relaxation component can be thus directly related to the sample ageing. Interestingly, the ageing of the other sample revealed slight changes as well: Fe2GluArgBH_o manifested itself by the same phases as Fe2GluArgBH_f, with the only difference being in their ratios. There is a small increase of Fe^3+^ content (of about 8 at.%) during two-years ageing, while, more importantly, a decrease in content of iron core-to-shell layers (from 63 at.% to 37 at.%) with a simultaneous increase of iron core component (from 16 at.% to 34 at.%). We hypothesize that the samples recrystallization took place during their ageing and is responsible for the observed results.

Last, but not least, the influence of l-amino acids addition order to ferric salt and subsequent chemical reduction by NaBH_4_ was also looked into. The as-prepared samples, labeled as Fe3ArgGluBH and Fe3GluArgBH, were characterized by Mössbauer spectroscopy (Figure 6e,f, respectively) revealing the phase composition which is listed in Table 1. The core-shell structures of Fe^0^-Fe_2_O_3_/FeOOH were generated in both samples. In addition, the Fe^0^ content was identical (within the experimental error) in both samples (around 38 at.%). This is quite opposite to the situation when ferrous salt was employed as the iron source (79 at.% of Fe^0^ in Fe2GluArgBH and 0 at.% of Fe^0^ in Fe2ArgGluBH). It can be thus concluded that an intermediate complex between Glu and ferrous sulphate (suggested in Scheme 1a), which is subsequently reduced by NaBH_4_, leads to the highest yield of Fe^0^. A more general declaration based on our results can be formulated as follows: ferrous and ferric salts complexed with two selected, subsequently added l-amino acids (Arg, Glu) revealed distinctly different behavior and, consequently, resulted in magnetic nanoparticles of various phase compositions. The importance of the choice and the order of the addition of the two selected l-amino acids are thus supporting evidence.

## 3. Experimental Section

### 3.1. Chemicals

Ferrous sulphate heptahydrate (Lachner, Brno, Czech Republic), ferric chloride hexahydrate (Lachner, Brno, Czech Republic), sodium borohydride (Sigma-Aldrich, Prague, Czech Republic), sodium hydroxide (Lachner, Brno, Czech Republic), l-arginine (abbreviated as Arg, Sigma-Aldrich, Prague, Czech Republic), l-glutamic acid (abbreviated as Glu, Sigma-Aldrich, Prague, Czech Republic), silver nitrate (Lachner, Brno, Czech Republic) were used as received from producers, *i.e.*, without any further purification. Deionized water was employed in all syntheses. Tetrahydrofuran (for UV spectroscopy, Fluka, Prague, Czech Republic) was employed for the preparation of samples for the characterization by TEM.

### 3.2. Syntheses of Iron Particles Mediated by a Single l-Amino Acid

A mixture (10 mL) of a particular l-amino acid (10 mM either Arg, or Glu) and FeSO_4_ (50 mM) and/or FeCl_3_ (50 mM) aqueous solution was quickly added into 40 mL of a cold aqueous solution of NaBH_4_ (0.15 M) under ambient conditions with vigorous stirring applied (900 rpm). After the formation of a precipitate, they were magnetically separated and/or centrifuged (9000 rpm for 15 min) if not revealing any magnetic response at room temperature. Then, the precipitates were dried under vacuum overnight in order to evaporate any residual solvent. The procedure yielded non-pyrophoric powders which were immediately sampled for the characterization by Mössbauer spectroscopy and SQUID (superconducting quantum interference device) magnetometry. The samples were labeled as Fe2GluBH (containing FeSO_4_ and Glu), Fe2ArgBH (containing FeSO_4_ and Arg), Fe3GluBH (containing FeCl_3_ and Glu), Fe3ArgBH (containing FeCl_3_ and Arg). Each type of sample was prepared at least three times to get statistically relevant data. As references, either ferrous sulphate mixed with a particular l-amino acid (named Fe2Glu for a mixture of ferrous salt with Glu and Fe2Arg for a mixture of ferrous salt with Arg), or a particular l-amino acid allowed to react with sodium borohydride (labeled as GluBH or ArgBH) were prepared (in the same concentrations as used in the syntheses of iron particles) and characterized by Mössbauer spectroscopy and/or SERS, respectively.

### 3.3. Syntheses of Iron Particles Mediated by Mixtures of l-Amino Acids

A mixture (10 mL) of FeSO_4_ (50 mM) and/or FeCl_3_ (50 mM) and the first l-amino acid (10 mM) aqueous solution was stirred for 5 minutes in order to completely dissolve both compounds. Then, the appropriate amount of the second l-amino acid (equal to the 10 mM concentration) was dissolved in the 10 mL mixture of ferrous sulphate and/or ferric chloride and the first l-amino acid. This mixture was again stirred for 5 min in order to completely dissolve the second l-amino acid. Finally, the mixture of both l-amino acids and ferrous sulphate and/or ferric chloride was quickly added into 40 mL of a cold aqueous solution of NaBH_4_ (0.15 mM) under ambient conditions with vigorous stirring applied (900 rpm). Isolation, drying and characterization of the precipitates were done exactly in the same way as in the case of systems containing a single l-amino acid (described above). The samples were named as (i) Fe2GluArgBH and Fe3GluArgBH for the sequence of Glu as the first and Arg as the second l-amino acid added; (ii) Fe2ArgGluBH and Fe3ArgGluBH for the sequence of Arg as the first and Glu as the second l-amino acid introduced.

### 3.4. Characterization by Mössbauer Spectroscopy

Each iron-containing dry sample (~10 mg) was firmly closed into a weighting paper and tightly covered by parafilm foil in order to avoid access of humidity and air. The transmission ^57^Fe Mössbauer spectra of 512 channels were collected using an in-laboratory developed MS-96 Mössbauer spectrometer [39] at a constant acceleration mode with a ^57^Co(Rh) source. Low-temperature Mössbauer spectra were recorded using a Spectromag (Oxford Instruments) cryomagnetic system, without the application of an external magnetic field. The isomer shift values were referred to α-Fe at room temperature.

### 3.5. Magnetization Measurements

SQUID (superconducting quantum interference device) magnetometer MPMS XL-7 (Quantum Design, San Diego, CA, USA) was used for the magnetization measurements, similarly as described in ref. [8]. The hysteresis loops were collected at a temperature of 5 and 300 K in external magnetic fields from −7 to +7 T. The zero-field-cooled (ZFC) and field-cooled (FC) magnetization curves were recorded on warming in the temperature range from 5 to 300 K and in an external magnetic field of 0.1 T after cooling in a zero magnetic field and a field of 0.1 T, respectively.

### 3.6. Characterization by X-Ray Powder Diffraction (XRD)

All X-ray diffraction patterns presented here were recorded on a PANalytical X’Pert PRO MPD diffractometer in Brag-Brentano geometry, equipped with an X’Celerator detector. The X-ray source produces iron filtered CoK_α_ radiation (λ = 0.178901 nm) which is adjusted by an incident beam Söller and anti-scatter slits, programmable divergence and diffracted beam anti-scatter slits. Samples were prepared on a zero-background Si slide and scanned in a 2θ range of 5–105°. The NIST (National Institute of Standard and Technology) standards SRM640 (Si) and SRM660 (LaB_6_) were used to evaluate the line positions and instrumental broadening. The diffraction patterns were processed employing the software High Score Plus (PANalytical) in conjunction with PDF4+ and ICSD databases (ISCD codes of identified phases are: α-Fe–76747, β-FeOOH–69606, and γ-FeOOH–108876).

### 3.7. Characterization by Transmission Electron Microscopy (TEM)

TEM images of selected samples were recorded on a JEOL JEM 2010 microscope equipped with a LaB_6_ cathode. A drop (4-μL) of a particular sample, highly diluted by tetrahydrofuran, was placed onto a holey-carbon copper grid and allowed to dry at room temperature. The samples were then measured by using accelerating voltage of 200 kV.

### 3.8. Synthesis of Silver Nanoparticles for Surface-Enhanced Raman Scattering Spectroscopy (SERS)

An aqueous solution (9 mL) of 2.2 mM of AgNO_3_ was added drop-wise into 75 mL of 1.1 mM aqueous solution of NaBH_4_ stirred in an Erlenmayer flask placed in an ice-bath. The ice-bath was removed five minutes after the addition of the last drop of silver nitrate solution. Stirring was continued for another 45 min to adjust the Ag colloid to laboratory temperature. Then, 10 min of heating at 40 °C was applied to the Ag colloid while stirring in order to facilitate the oxidation of any non-reacted borohydride. Finally, the Ag colloid was allowed to cool down to laboratory temperature with continuous stirring. The resulting Ag colloid was yellow in color, with the maximum of surface plasmon extinction at 390 nm, containing particles of ~12 nm in diameter. It was stable for at least 3 months without addition of any stabilizers if stored in the dark at room temperature.

### 3.9. UV-Vis and SERS Spectral Measurements

A solution (10 μL) of a particular l-amino acid (10 mM), or ArgBH/GluBH solution (1% wt) were introduced into 1 mL of Ag colloid. UV-Vis spectra were recorded on a double-beam Perkin-Elmer spectrometer in a 1 cm cuvette. SERS spectra were measured on a Horiba Jobin Yvon Raman spectrometer in backscattering mode, using macro-sampler for a 1 cm cuvette, employing the 632.8 nm excitation wavelength, 100 μm slit and 50× objective. Each SERS spectrum was accumulated 20 times for 10 s, baseline corrected and presented without any smoothing.

## 4. Conclusions

To sum up, the choice and order of l-amino acids applied in the synthesis can intentionally drive the reaction either towards magnetically strong Fe^0^-Fe_2_O_3_ core-shell particles with surface modification by l-amino acids, useful for an efficient removal of halogenated organic compounds (e.g., trichloroethane) as demonstrated in [8]; or toward superparamagnetic Fe_2_O_3_ particles applicable in magnetic resonance imaging where the surface modification by a biocompatible compound is of crucial importance. Taking into account TEM images, Mössbauer and SQUID data, there is a distinct difference in the order of l-amino acids addition to ferrous salt on the resulting iron-containing nanoparticulate structures. Due to an intermediate complex formation between Glu and ferrous sulphate, which is subsequently reduced by NaBH_4_, the highest yield of Fe^0^ is evidenced in magnetically strong core-shell particles stemming from this reaction mixture. Over two years of ageing of these nanoparticles (prepared in amino acid mixtures), partial recrystallization takes place.

## Figures and Tables

**Table 1:** Values of the Mössbauer hyperfine parameters, derived from the Mössbauer spectra of the Fe2ArgBH, Fe2GluBH, Fe2pH10BH, Fe3ArgBH, Fe3GluBH, Fe2Arg, Fe2Glu, Fe2ArgGluBH_f (for fresh sample), Fe2ArgGluBH_o (for two-years aged sample), Fe2GluArgBH_f (for fresh sample), Fe2GluArgBH_o (for two-years aged sample), Fe3ArgGluBH, and Fe3GluArgBH sample, where *T* is the temperature of measurement, *B*_ext_ is the induction of external magnetic field, δ is the isomer shift, Δ*E_Q_* is the quadrupole splitting, *B*_hf_ is the hyperfine magnetic field, *B*_eff_ is the effective hyperfine magnetic field (*i.e.*, a vector sum of *B*_hf_ and *B*_ext_), and RA is the relative spectral area of individual components.

Sample	T (K)	*B*_ext_ (T)	Component	δ ± 0.01 (mm/s)	Δ*E_Q_* ± 0.01 (mm/s)	*B*_hf_ ± 0.3 (T)	*B*_eff_ ± 0.3 (T)	RA ± 1 (%)	Assignment
Fe2ArgBH	300	0	Doublet	0.35	0.79	-	-	40	Fe^3+^ iron oxide–superparamagnetic state
			Sextet	0.34	0.02	36.4	-	60	Fe^3+^ iron oxide–blocked state
	5	5	Sextet	0.39	−0.03	-	51.3	52	γ-Fe_2_O_3_–tetrahedral sites
			Sextet	0.48	−0.02	-	46.9	38	γ-Fe_2_O_3_–octahedral sites
			Sextet	0.10	0.00	-	32.4	10	Iron

Fe2GluBH	300	0	Doublet	0.36	0.78	-	-	14	Fe^3+^ iron oxide/oxyhydroxide shell
			Sextet	0.00	0.00	32.8	-	86	Iron core

Fe2pH10BH	300	0	Doublet	0.35	0.70	-	-	100	Fe^3+^ iron oxide/oxyhydroxide

Fe3ArgBH	300	0	Doublet	0.36	0.75	-	-	57	Fe^3+^ iron oxide/oxyhydroxide shell
			Sextet	0.01	−0.02	30.4 *	-	43	Iron core

Fe3GluBH	300	0	Doublet	0.36	0.74	-	-	20	Fe^3+^ iron oxide/ oxyhydroxide
			Sextet	0.00	0.00	33.1	-	80	Iron core

Fe2Arg	300	0	Doublet	1.26	3.19	-	-	38	Fe^2+^ component
			Doublet	0.37	0.56	-	-	62	Fe^3+^ component

Fe2Glu	300	0	Doublet	1.26	3.18	-	-	87	Fe^2+^ component
			Doublet	0.39	0.43	-	-	13	Fe^3+^ component

Fe2ArgGluBH_f	300	0	Doublet	0.31	0.70	-	-	52	Fe^3+^ iron oxide/ oxyhydroxide–superparamagnetic state
			Sextet	0.31	0.00	30.6	-	48	Fe^3+^ iron oxide/ oxyhydroxide–relaxation component

Fe2ArgGluBH_o	300	0	Doublet	0.35	0.75	-	-	55	Fe^3+^ iron oxide/ oxyhydroxide superparamagnetic state
			Singlet	0.35	-	-	-	45	Fe^3+^ iron oxide/ oxyhydroxide–relaxation component

Fe2GluArgBH_f	300	0	Doublet	0.36	0.77	-	-	21	Fe^3+^ iron oxide/ oxyhydroxide shell
			Sextet	0.01	0.00	32.5	-	16	Iron core
			Sextet	0.04	−0.05	30.1 *	-	63	Iron core-to-shell layers

Fe2GluArgBH_o	300	0	Doublet	0.36	0.79	-	-	29	Fe^3+^ iron oxide/ oxyhydroxide shell
			Sextet	0.00	0.00	33.1	-	34	Iron core
			Sextet	0.00	0.00	25.2 *	-	37	Iron core-to-shell layers

Fe3ArgGluBH	300	0	Doublet	0.36	0.75	-	-	61	Fe^3+^ iron oxide/ oxyhydroxide shell
			Sextet	0.00	−0.03	32.9	-	39	Iron core

Fe3GluArgBH	300	0	Doublet	0.37	0.69	-	-	63	Fe^3+^ iron oxide/ oxyhydroxide shell
			Sextet	0.01	−0.04	32.9	-	37	Iron core

* The average value of the hyperfine magnetic field derived from distribution of the hyperfine magnetic field.
